# Automated segmentation of pituitary adenomas, pituitary gland, and internal carotid arteries on routine coronal contrast-enhanced T1-weighted MRI: a single-sequence feasibility study

**DOI:** 10.3389/fendo.2026.1851379

**Published:** 2026-06-30

**Authors:** Woojae Hong, Ho Kang, Jong Ha Hwang, Seong-Min Kim, Yong Hwy Kim, Hyunggun Kim

**Affiliations:** 1 Department of Biomechatronic Engineering, Sungkyunkwan University, Suwon, Gyeonggi, Republic of Korea; 2 Department of Neurosurgery, Seoul National University Bundang Hospital, Seoul National University College of Medicine, Seongnam, Republic of Korea; 3 Department of Neurosurgery, Seoul National University Hospital, Seoul National University College of Medicine, Seoul, Republic of Korea

**Keywords:** deep learning, internal carotid artery, magnetic resonance image, pituitary adenoma, pituitary gland

## Abstract

**Introduction:**

Pituitary adenomas (PAs) alter the anatomy of the pituitary gland (PG) and internal carotid arteries (ICAs), requiring accurate segmentation for surgical planning and risk assessment. Multi-sequence magnetic resonance imaging (MRI)-based automatic segmentation methods have been employed but remain limited in routine clinical practice due to protocol inconsistencies. This study aimed to evaluate the feasibility and clinical relevance of Swin-Unet and nnU-Net for automated segmentation of the PA, PG, and ICA using only contrast-enhanced T1-weighted (T1CE) coronal MRI.

**Methods:**

A retrospective cohort of 255 patients with surgically confirmed PAs was analyzed. T1CE coronal MRI, acquired from heterogeneous scanners and protocols, was used for model development. Manual annotations of the PA, PG, and ICA by expert neurosurgeons served as ground truth. Two deep learning architectures were evaluated: 1) Swin-Unet, a transformer-based U-Net variant, and 2) nnU-Net, a self-configuring CNN framework. Models were trained using standard preprocessing and five-fold cross-validation, with ensemble predictions generated for final evaluation. Model performance was assessed using the Dice similarity coefficient (DSC), 95th percentile Hausdorff distance (HD95), true-positive rate (TPR), and false-positive rate (FPR). Additionally, slice-level presence detection and per-patient processing time were evaluated.

**Results:**

Both models demonstrated comparable voxel-wise performance, with mean DSC = 0.70 (95% CI: 0.66-0.74) for Swin-Unet and 0.69 (95% CI: 0.65-0.73) for nnU-Net. Segmentation accuracy was lower for the PG compared to the PA and ICA. Boundary-based evaluation showed a mean HD95 of 5.21 mm (95% CI: 4.40-6.03) for Swin-Unet and 5.30 mm (95% CI: 4.44-6.16) for nnU-Net. Slice-level recognition yielded a mean F1-score of 0.89 ± 0.09 for Swin-Unet and 0.87 ± 0.08 for nnU-Net across the PA, PG, and ICA, respectively. The mean computation time was 78.4 seconds per patient (95% CI: 61.5–95.5), and the segmentation outputs facilitated approximate 3D reconstructions for qualitative assessment.

**Discussion:**

Our findings demonstrate the feasibility of automated segmentation of the PA, PG, and ICA using single-sequence T1CE coronal MRI. While voxel-level accuracy was modest, the combination of reliable slice-level recognition, rapid processing, and 3D visualization suggests potential utility as an adjunct tool for radiologic assessment and surgical planning.

## 1 Introduction

Pituitary adenomas (PAs) are currently classified as pituitary neuroendocrine tumors (PitNETs) in the 2022 World Health Organization (WHO) classification (1). PAs account for 10–15% of all brain tumors, representing one of the most common intracranial neoplasms (2). Depending on size, location, and secretory activity, these tumors produce a variety of clinical manifestations, including visual field impairment, endocrine dysfunction, and headache (3). Accurate characterization of the spatial relationship between PAs and surrounding critical structures, including the pituitary gland (PG), internal carotid arteries (ICAs), and cavernous sinuses, is clinically imperative (4). In particular, assessment of cavernous sinus invasion and determination of the tumor-ICA interface are key factors in preoperative risk stratification and surgical decision-making (5).

Although pituitary magnetic resonance imaging (MRI) provides the anatomical basis for diagnosis and surgical planning, routine sellar MRI protocols are largely based on thin-section coronal and sagittal two-dimensional (2D) image series (4, 6). Comprehensive understanding of the three-dimensional (3D) spatial relationship among the PA, PG, and ICA allows for more precise determination of cavernous sinus invasion, improved assessment of tumor proximity to critical vascular structures, and clearer delineation of surgical corridors (7). Unlike conventional 2D imaging, 3D visualization provides superior representation of distorted sellar anatomy, facilitates preoperative planning, and enables integration with navigation systems or patient education, collectively demonstrating significant clinical utility in pituitary surgery (8).

MRI remains the cornerstone of PA diagnosis and preoperative assessment. In clinical practice, however, MRI acquisition protocols exhibit substantial variability across institutions, scanner platforms, and even individual examinations depending on diagnostic objectives (9). Such variability poses challenges for obtaining datasets with standardized coverage, slice thickness, and contrast parameters. Previous segmentation studies have reported moderate to reasonable accuracy using multi-sequence and multi-planar MRI, yet these investigations typically employed data acquired under uniform protocols, limiting their applicability to routine clinical practice (10–12). Consistent acquisition of multiple MRI sequences across diverse clinical settings is rarely feasible, and segmentation performance is expected to decline when applied to heterogeneous imaging data (13).

Contrast-enhanced T1-weighted (T1CE) coronal MRI is routinely acquired in nearly all patients with suspected pituitary pathology and provides reliable visualization of the PA, PG, and ICA (14). This sequence is indispensable for evaluating cavernous sinus invasion, determining Knosp grade, and planning surgical approach, often serving as the primary imaging dataset for operative decision-making (15). Despite potentially lower performance metrics relative to multi-sequence models, single-sequence segmentation offers distinct advantages in clinical applicability and translational feasibility, representing a valuable practical tool for bridging imaging research with routine clinical practice.

Deep learning (DL) techniques have established strong foundations for medical image segmentation, with U-Net and nnU-Net (16) serving as reference architectures, and transformer-based models, such as the Swin-Transformer (17), offering improved capacity to capture global image context. Automated segmentation of the PA, PG, and ICA on T1CE coronal MRI has the potential to support evaluation of cavernous sinus invasion, ICA proximity, and preoperative surgical planning. Even moderate segmentation accuracy can provide clinically meaningful insights through reliable structure identification and approximate 3D reconstructions. In this study, Swin-Unet (18) and nnU-Net (16) were applied to T1CE coronal MRI acquired with heterogeneous scanners and imaging protocols to evaluate the feasibility and clinical value of a single-sequence segmentation approach.

## 2 Methods

### 2.1 Patient selection and allocation

This retrospective study was approved by the Institutional Review Board of Seoul National University Hospital (No. 2008-027-1145) and conducted in compliance with the Declaration of Helsinki. Written consent was waived for this retrospective study. Clinical, pathological, and radiological information of 255 patients who underwent endoscopic skull base surgery for PA at Seoul National University Hospital from March 2010 to August 2020 were retrospectively reviewed through electronic medical and radiological records. All cases were pathologically confirmed as PA. The dataset was divided into a training set (218 patients, 85.5%) and an independent held-out test set (37 patients, 14.5%). The held-out test set was not involved in any stage of model training, validation, model selection, hyperparameter tuning, or ensemble construction. Five-fold cross-validation was conducted exclusively within the training set, and the final ensemble models were evaluated only on the held-out test set.

### 2.2 MRI acquisition and manual segmentation

Preoperative MRI of the sellar turcica and parasellar region was conducted for all patients using 1.5T or 3T scanners with T1-and T2-weighted spin echo sequences before and after administration of Godoberi (Gadovist, Bayer) contrast medium (19). MRI scans were collected from 255 patients using five different scanner models: Magnetom Skyra and Magnetom Skyra FIT (Siemens Healthineers, Erlangen, Germany), Ingenia CX (Philips Healthcare, Best, The Netherlands), and Discovery MR750w and Signa HDxt (GE Healthcare, Milwaukee, WI, USA). Detailed acquisition parameters were as follows: echo time of 11.4 ms [10.5–12.4], flip angle of 74.9° [70.6–79.3], bandwidth of 159.4 Hz/pixel [147.9–171.0], slice number of 18.5 [16.2–20.9], echo train length of 1.90 [1.51–2.28], repetition time of 397 ms [365–429], and acquisition type of 2D. Complete patient-level acquisition parameters are provided in the **Supplementary Material** (.csv file). T1CE was selected for its superior delineation of tumor boundaries and cavernous sinus invasion. Coronal sections were utilized due to their routine clinical use and enhanced depiction of tumor invasion into surrounding structures, particularly the cavernous sinus. A retrospective, independent MRI review was conducted by two neurosurgeons (YHK and HK) with 24 and 12 years of clinical experience, respectively. Both investigators have extensive experience in PA surgery (over 2, 000 and 500 cases, respectively) and routinely interpret preoperative MRI as part of surgical planning. The following parameters were assessed: (1) cavernous sinus invasion and Knosp grade, (2) tumor consistency (solid or cystic, with cystic tumors defined as those with >50% cystic composition), (3) tumor size classification (macro- or microadenoma, with macroadenoma defined as >1 cm in maximal diameter), and (4) tumor volume (T1CE coronal MRI) (20). Tumor volume was calculated by integrating the segmented tumor areas across all coronal slices, multiplied by the given slice thickness.

Manual annotation was performed on T1CE coronal images using ITK-SNAP 4.0.2 (21). Prior to manual annotation, a standardized segmentation protocol was established by the two neurosurgeons to define the target structures and to minimize ambiguity in boundary delineation. The PA mask encompassed the entire visible tumor extent on T1CE coronal images, including intratumoral cystic components when present. The PG mask included visually identifiable residual PG tissue. The ICA mask was conservatively defined to include only the sellar and parasellar ICA segments within the coronal T1CE MRI coverage that were clinically relevant for assessing tumor–ICA relationship and determining the presence of cavernous sinus invasion. Distal ICA segments beyond this target region were excluded. In cases with severe ICA compression, distortion, or partial obscuration, only confidently visualized and anatomically traceable segments were included. The two neurosurgeons independently delineated regions of interest, including the PA, PG, and ICA. Each segmentation was subsequently reviewed and verified by the other neurosurgeon, and discrepancies were resolved through iterative consensus discussion. The resulting consensus annotations served as the reference standard for model training and evaluation. Quantitative inter-rater and intra-rater variability analyses were not performed in the present study.

### 2.3 Clinical data acquisition

Clinical information of the patients, including age, gender, symptoms, visual field, and adenohypophyseal hormonal status, was collected. The anterior pituitary function was evaluated preoperatively in all patients according to the protocol described in our previous studies (19, 22, 23). Adrenocorticotropic hormone (ACTH) deficiency was diagnosed when the peak cortisol level was <18 µg/dL with a low-to-normal serum ACTH concentration in the short Synacthen test. Thyroid-stimulating hormone (TSH) deficiency was diagnosed when serum free thyroxine concentration level was <0.7 ng/dL with a low-to-normal serum TSH level. Hypopituitarism was defined as a deficiency in at least one anterior pituitary hormone. Pathological evaluation included the final diagnosis, Ki-67 index, and immunohistochemical staining results for each anterior pituitary hormone. Summarized clinical, pathological, and radiological characteristics are presented in **Table 1**. Complete patient-level clinical data are not provided in the **Supplementary Material** due to the institutional privacy regulations protecting confidential patient health information.

**Table 1:** Baseline clinical, pathological, and radiological characteristics of the patients.

	Overall (n=255)	Training dataset (n=218)	Held-out test dataset (n=37)	*p*
Male, n	122 (47.8%)	99 (45.4%)	23 (62.2%)	0.09
Age, years	49.7±14.7	50.7±14.2	43.8±16.5	0.008
Visual field defect, n	152 (73.8%)	136 (74.3%)	16 (69.6%)	0.81
Anterior pituitary hormone deficiency, n	105 (60.7%)	86 (58.5%)	19 (73.1%)	0.24
Clinical diagnosis, n				0.97
Non-functioning adenoma	190 (74.5%)	163 (74.8%)	27 (73.0%)	
Acromegaly	42 (16.5%)	36 (16.5%)	6 (16.2%)	
Cushing disease	14 (5.5%)	11 (5.0%)	3 (8.1%)	
Prolactinoma	8 (3.1%)	7 (3.2%)	1 (2.7%)	
TSHoma	1 (0.4%)	1 (0.5%)	0 (0.0%)	
PitNET classification, n^*^				0.05
Corticotroph	41 (16.1%)	35 (16.1%)	6 (16.2%)	
Somatotroph	41 (16.1%)	32 (14.7%)	9 (24.3%)	
Lactotroph	29 (11.4%)	27 (12.4%)	2 (5.4%)	
Thyrotroph	5 (2.0%)	3 (1.4%)	2 (5.4%)	
Gonadotroph	48 (18.8%)	37 (17.0%)	11 (29.7%)	
Plurihormonal	9 (3.5%)	9 (4.1%)	0 (0.0%)	
Null cell	82 (32.2%)	75 (34.4%)	7 (18.9%)	
Ki-67 index, %	1.8 (1.0, 3.1)	1.6 (1.0, 2.9)	3.0 (1.6, 4.2)	0.003
Radiological characteristics				
Microadenoma, n	18 (7.1%)	15 (6.9%)	3 (8.1%)	1.00
Tumor volume, cm^3^	6.2 (3.1, 10.6)	6.2 (3.1, 11.1)	5.8 (2.3, 8.2)	0.35
Knosp grade, n				0.37
0	35 (13.4%)	34 (15.6%)	1 (2.7%)	
1	75 (29.4%)	60 (27.5%)	15 (40.5%)	
2	69 (27.1%)	59 (27.1%)	10 (27.0%)	
3A	54 (21.2%)	46 (21.1%)	8 (21.6%)	
3B	4 (1.6%)	3 (1.4%)	1 (2.7%)	
4	18 (7.1%)	16 (7.3%)	2 (5.4%)	
Cystic tumor, n	102 (40.2%)	92 (42.4%)	10 (27.0%)	0.11

Continuous variables were presented as mean ± standard deviation or median with interquartile range according to the normality tested by the Shapiro-Wilk test.

^*^ Immunohistochemical staining-based diagnosis according to WHO classification 2021.

PitNET, pituitary neuroendocrine tumor.

### 2.4 Model development

Two architectures were implemented for automated segmentation of the PA, PG, and ICA on T1CE coronal MRI. Swin-Unet (18), a transformer-based variant of the U-Net, integrates hierarchical self-attention mechanisms within an encoder–decoder structure. This design allows the model to capture long-range contextual dependencies while preserving local feature representation, which is particularly valuable for segmenting the anatomically complex and sellar and parasellar regions. In contrast, nnU-Net (16) represents a convolutional neural network (CNN)–based framework widely regarded as a benchmark in medical image segmentation. Its self-configuring design automatically adapts preprocessing, network architecture, and training strategies to dataset-specific characteristics, offering a robust and generalizable baseline for performance comparison with transformer-based methods. Detailed architectural specifications for both models are provided in the **Supplementary Figures 1**, **2**.

Image processing was performed in four steps. The first step involved applying N4 bias field correction to the T1CE coronal MR scans utilizing N4ITK (24). Next, intensity normalization was conducted using Z-Score (25) using the Python intensity-normalization package (26). For Swin-Unet, additional fixed external preprocessing was applied to ensure uniform input dimensions. Specifically, images were resampled to a fixed in-plane spatial resolution of 0.3 × 0.3 mm and the central sellar region was cropped to 256 × 256 pixels. In contrast, nnU-Net employed its standard self-configuring preprocessing pipeline, which automatically optimized resampling, cropping, and normalization procedures based on dataset characteristics. This methodological distinction reflects the fundamental differences in preprocessing requirements between the two architectures.

Model training was performed using a five-fold cross-validation strategy. The model exhibiting the lowest validation loss was selected for subsequent analysis. Final predictions on the test set were generated using an ensemble approach to enhance robustness and generalizability when applied to heterogeneous MRI data. All experiments were conducted on an identical workstation equipped with an Nvidia Titan RTX GPU (24 GB memory) and Intel^®^ Core™ i9-10940X CPU. All training hyperparameters followed the default configuration of each model.

### 2.5 Evaluation metrics

Performance was assessed at three levels: voxel-wise segmentation (Dice similarity coefficient; DSC, true-positive rate; TPR, and false-positive rate; FPR), boundary accuracy (95th percentile Hausdorff distance; HD95) and slice-level recognition (accuracy, precision, recall, specificity, and F1-score). For slice evaluation, any voxel overlap between prediction and ground truth was counted as a true positive, and false detections were manually verified. This complementary measure captures the clinical relevance of structured recognition and spatial relationships beyond voxel-level segmentation.

The end-to-end processing time was measured for each case, including preprocessing (data loading, N4 bias correction, Z-score normalization, resampling, and cropping) and model inference. The mean processing time per patient was calculated as an indicator of workflow efficiency. To minimize potential interference from background processes, each measurement was repeated five times using the identical hardware, and the average value was recorded.

### 2.6 Statistical analysis

All statistical analyses were performed using the R-based Python SciPy Statistical function module (version 1.13.1) and statsmodels module (version 0.14.1) (27). For continuous variables, normality was tested using the Shapiro-Wilk test. Data were presented as mean ± standard deviation or median (interquartile range [IQR]) depending on the p values. To compare the segmentation performance of the Swin-Unet and nnU-Net models, statistical analyses were conducted on DSC, HD95, TPR, and FPR values derived from the test set. The output of each model was evaluated at both the whole-structure level (mean across three classes) and class-wise (PA, PG, and ICA). Paired DSC, HD95, TPR, and FPR values were assessed using either paired t-tests or Wilcoxon signed-rank tests, depending on the normality of the paired differences (determined by the Shapiro–Wilk test). Effect sizes were computed using Cohen’s d for normally distributed data, and Wilcoxon r for non-normal distributions. Bonferroni correction was applied for multiple comparisons, with statistical significance defined as adjusted p < 0.05.

Subgroup analyses were additionally conducted for exploratory purposes using structured clinical metadata. As no formal hypothesis was specified as *a priori*, unadjusted p-values are reported. Interpretation was primarily based on effect sizes and consistency of trends rather than strict statistical significance. Clinical characteristics included Knosp grade (0–4), T1 and T2 MRI signal intensity (low/iso/high), immunohistochemistry (IHC) profiles (GH, ACTH, FSH, LH, etc.), and diagnosis categories such as acromegaly or microadenoma. For each subgroup with ≥ 3 samples, DSCs from the two models were compared using paired statistical tests. In clinically relevant subgroups (e.g., acromegalic patients, GH-positive tumors), the paired DSC values from the two models were evaluated using paired t-tests when the normality of the paired differences was confirmed by the Shapiro–Wilk test (p > 0.05), and Wilcoxon signed-rank tests otherwise. For each comparison, the effect size was calculated: Cohen’s d for normally distributed differences and Wilcoxon r for non-parametric comparisons.

## 3 Results

### 3.1 Clinical, pathological, and radiological characteristics of the patients

The clinical, pathological, and radiological information of the patients with PA is summarized in **Table 1**. The mean patient age was 49.7 years, with 122 males (47.8%). Visual field defects and anterior pituitary hormonal deficiencies were observed in 152 (73.8%) and 105 patients (60.7%), respectively. The most common clinical diagnosis was non-functioning PAs, affecting 190 individuals (74.5%). Immunohistochemical analysis revealed that null cell tumor represented the most frequently occurring subtype (82/255, 32.2%). On T1-weighted imaging, the most common tumor signal was iso-intensity (197/255, 77.3%), while hyperintensity was the most common on T2-weighted imaging (175/255, 68.6%). A majority (92.9%) of tumors were classified as macroadenomas. The median tumor volume was 6.4 cm^3^, with cystic tumors identified in 102 patients (40.2%). Cavernous sinus invasion was observed in 76 patients (29.8%). There were statistically significant differences in age (50.7 ± 14.2 vs. 43.8 ± 16.5 years, *p* = 0.008) and the Ki-67 index [1.6 (IQR, 1.0–2.9) vs. 3.0 (IQR, 1.6–4.2), *p* = 0.003] between the training and test datasets.

**Figure 1:**
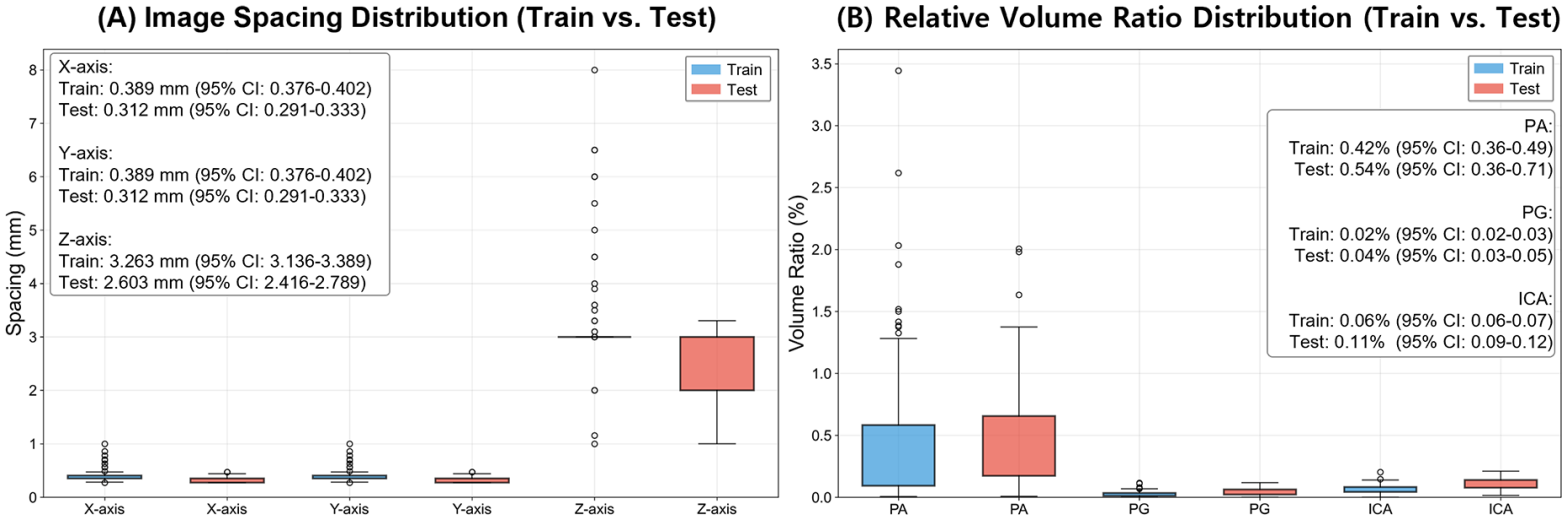
Overall distributions of image resolution and relative volume ratio for both the training and held-out test data. **(A)** Voxel spacing distribution in mm for all three spatial axes. The x- and y-axes represent in-plane resolution, whereas the z-axis represents through-plane slice spacing. **(B)** Relative volume ratio distribution of all three classes (PA, PG, and ICA) with respect to the total MRI volume. PA, pituitary adenoma; PG, pituitary gland; ICA, internal carotid artery.

The average in-plane image resolution was 0.39 mm [95% CI: 0.38-0.41], with a corresponding matrix size of 488 [457-501] × 487 [474-500] and a slice thickness of 2.49 mm [2.37-2.61]. Volume analysis demonstrated that PAs accounted for 0.44% [0.38–0.50] of the total intracranial volume, while PG and ICA comprised 0.03% [0.02–0.03] and 0.07% [0.06–0.07], respectively. The overall distributions of image resolution and relative volume ratio are shown in **Figure 1**.

### 3.2 Voxel-wise and boundary-based segmentation performance

**Figure 2:**
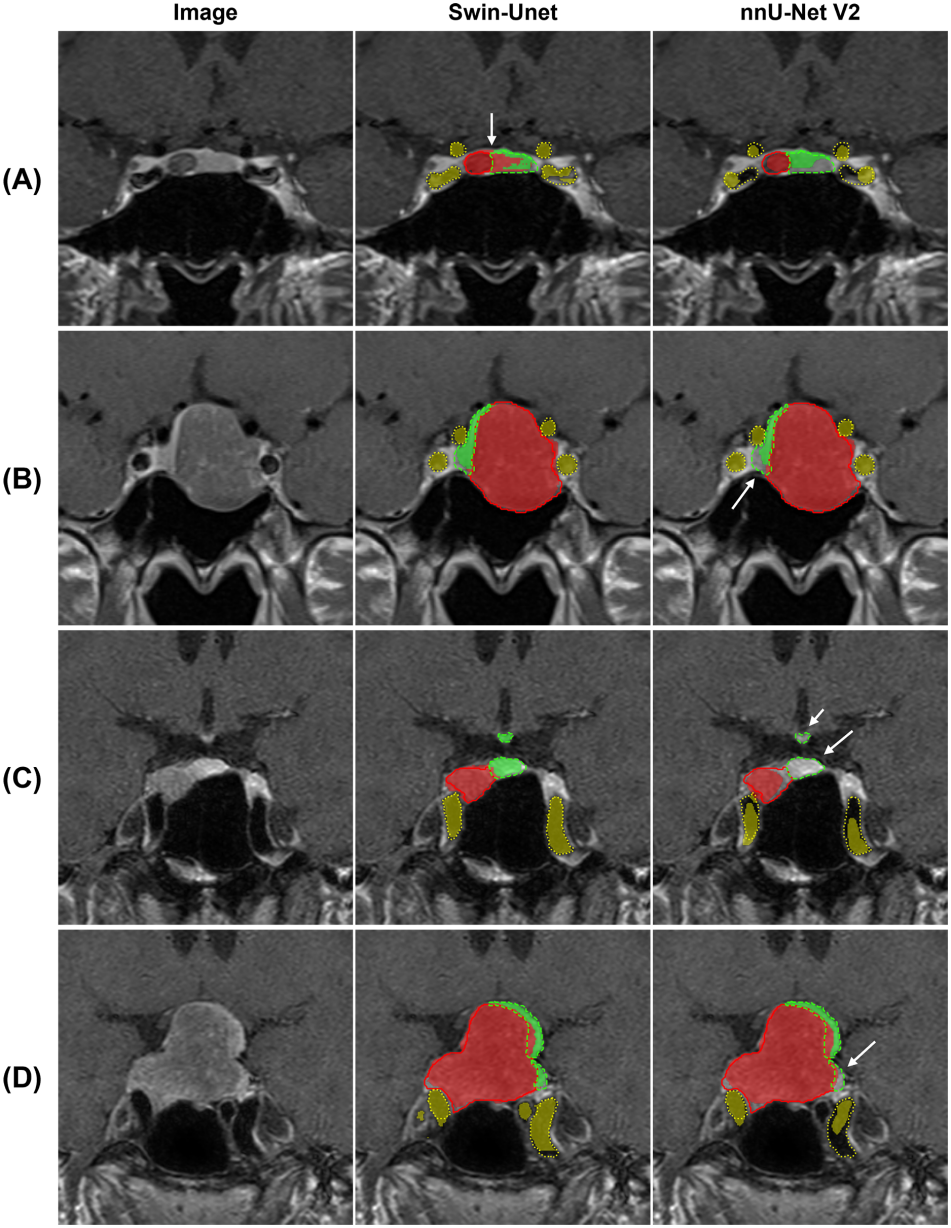
Representative segmentation outcomes of PA, PG, and ICA from the held-out test dataset corresponding to Knosp grade 1, 2, and 3A. PA, PG, and ICA are visualized in red, green, and yellow, respectively. Ground truth annotations are presented as corresponding color-coded contours: solid lines for PA, dashed lines for PG, and dotted lines for ICA. **(A)** Microadenoma with Knosp grade 1. **(B)** Acromegaly with Knosp grade 1. **(C)** Acromegaly with Knosp grade 2. **(D)** Acromegalic appearance with Knosp grade 3A. The white arrows indicate regions corresponding to classes that showed differences. DSC, Dice similarity coefficient; ICA, internal carotid artery; PA, pituitary adenoma.

The nnU-Net model was trained using Z-score normalized images in both 2D and 3D full-resolution settings. The final output was generated through ensemble integration of the 2D and 3D model predictions. A summary of the performance metrics, evaluated using ensemble of the five models derived from five-fold cross-validation for both Swin-Unet and nnU-Net, is presented in **Table 2**. Compared to the nnU-Net model, the Swin-Unet model demonstrated higher DSCs of 0.702, 0.560, and 0.716 for the mean of three classes, PG, and ICA, respectively. However, these differences were not statistically significant (p = 1.000, 1.000, and 1.000). Across the three target structures, Swin-Unet showed a mean HD95 of 5.21 mm [95% CI: 4.40–6.03], while nnU-Net achieved a mean HD95 of 5.30 mm. No statistically significant difference in overall HD95 was found between the two models (p = 1.000). The nnU-Net model outperformed the Swin-Unet model in terms of TPR for the mean of three classes and ICA (p < 0.05), while no statistically significant differences were observed for PG and PA. In contrast, the Swin-Unet model exhibited lower FPR values than the nnU-Net model. However, these differences were not statistically significant (p = 1.000, 1.000, and 1.000). **Figure 2** presents several representative cases corresponding to Knosp grade 1, 2, and 3A.

**Table 2:** Segmentation performance (DSCs, HD95, TPR, and FPR) of ensemble of the five models for both Swin-Unet and nnU-Net.

Mean [95% CI]	DSCs	HD95 (mm)	TPR	FPR
	Swin-Unet			
Mean of three classes	0.70 [0.66-0.74]	5.21 [4.40-6.03]	0.70 [0.65-0.74]	0.002 [0.002-0.003]
PA (class 1)	0.83 [0.78-0.88]	3.34 [2.34-4.35]	0.85 [0.81-0.89]	0.003 [0.002-0.005]
PG (class 2)	0.56 [0.50-0.63]	6.33 [4.50-8.17]	0.53 [0.46-0.61]	0.001 [0.001-0.002]
ICA (class 3)	0.72 [0.66-0.77]	6.08 [4.51-7.65]	0.70 [0.64-0.77]	0.002 [0.001-0.002]
	nnU-Net			
Mean of three classes	0.69 [0.65-0.73]	5.30 [4.44-6.16]	0.78 [0.76-0.84]	0.003 [0.002-0.003]
*p* value	1.00	1.00	<0.05	1.00
PA (class 1)	0.84 [0.80-0.88]	2.64 [2.07-3.20]	0.89 [0.83-0.94]	0.004 [0.002-0.006]
*p* value	1.00	0.55	0.53	1.00
PG (class 2)	0.54 [0.46-0.61]	6.50 [4.27-8.72]	0.68 [0.59-0.77]	0.001 [0.0007-0.001]
*p* value	1.00	0.86	0.08	1.00
ICA (class 3)	0.70 [0.66-0.75]	6.91 [5.54-8.27]	0.84 [0.79-0.89]	0.002 [0.001-0.003]
*p* value	1.00	0.13	<0.05	1.00

**Figure 3:**
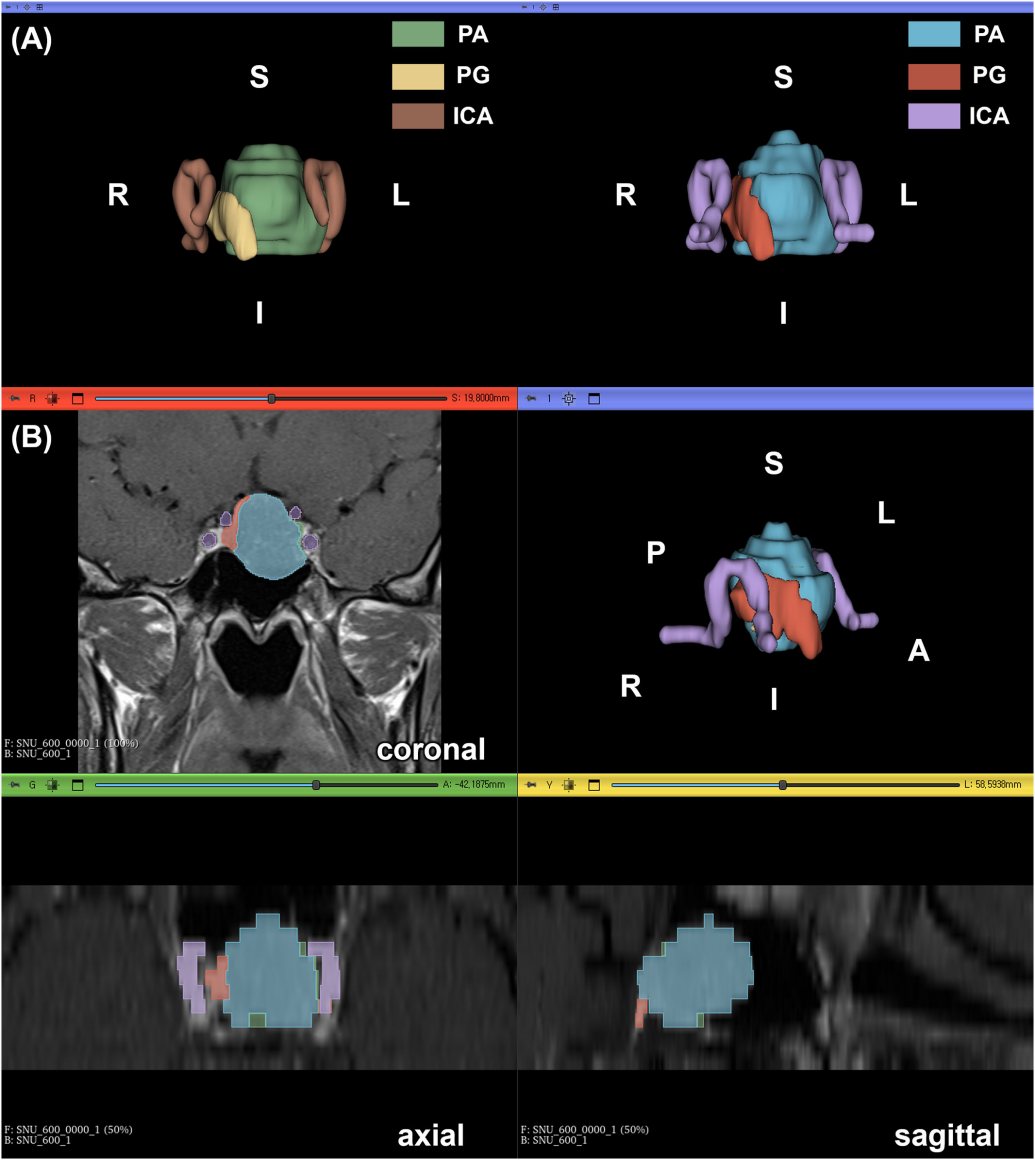
Representative 3D visualizations of the best performing case obtained directly from 3D Slicer. The best performing case was identified by the highest mean DSC across the three target structures (PA, PG, and ICA). This case showed a mean DSC of 0.84, with DSCs of 0.94 for PA, 0.71 for PG, and 0.86 for ICA. **(A)** Surface-rendered 3D visualization generated in 3D Slicer, showing manual reference segmentations (left) and model predictions (right). Anatomical orientation markers indicate superior (S), inferior (I), right (R), and left (L). **(B)** Multiplanar and oblique views showing segmentation masks overlaid on the T1CE MRI volume. The axial and sagittal images are multiplanar reconstructions generated from the original coronal T1CE volume with the corresponding segmentation masks in 3D Slicer, not overlays on independently acquired axial or sagittal MRI sequences. PA, pituitary adenoma; PG, pituitary gland; ICA, internal carotid artery; DSC, Dice similarity coefficient; T1CE, contrast-enhanced T1-weighted.

**Figure 4:**
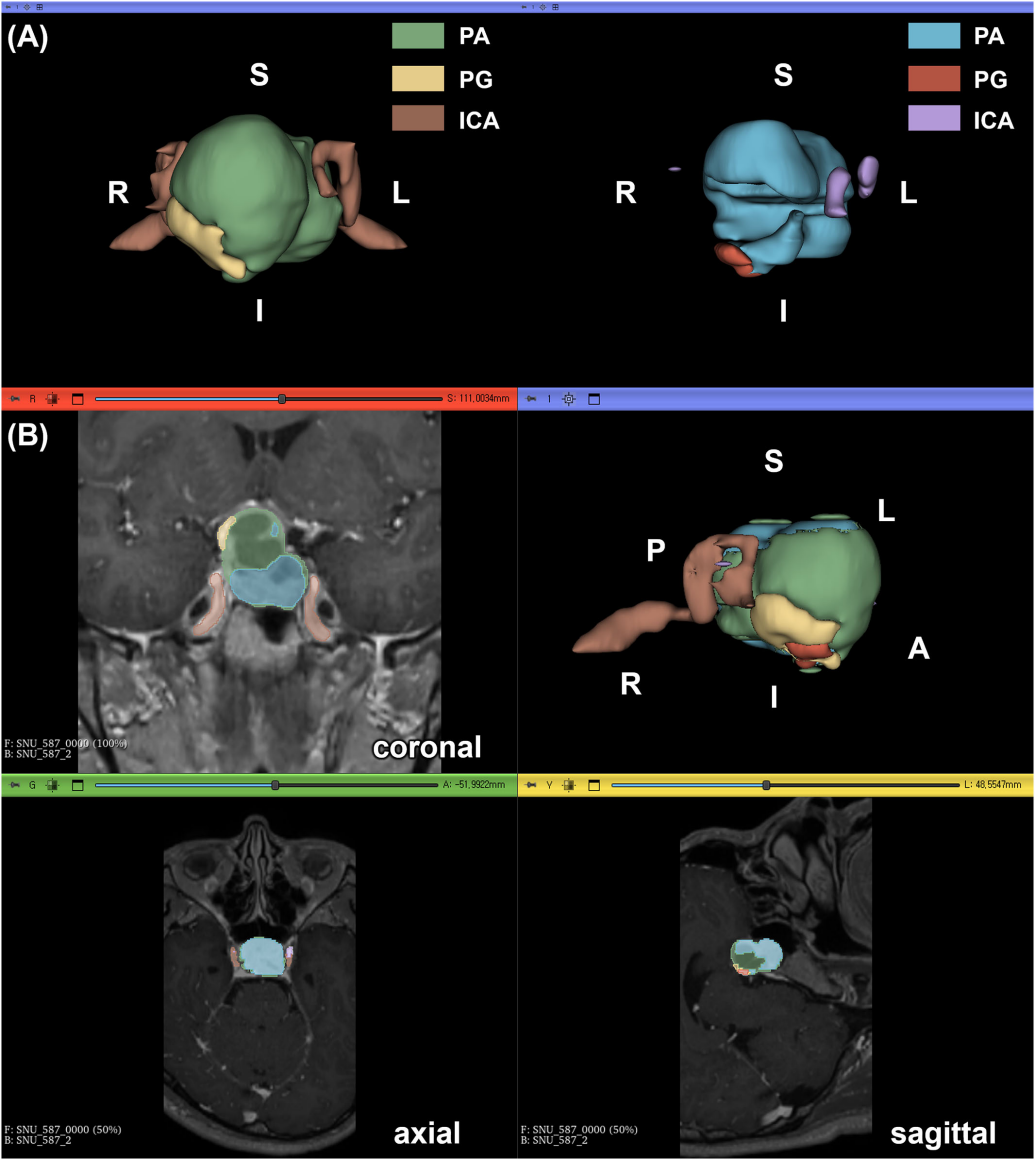
Representative 3D visualizations of the worst performing case obtained directly from 3D Slicer. The worst performing case was identified by the lowest mean DSC across the three target structures (PA, PG, and ICA). This case showed a mean DSC of 0.41, with DSCs of 0.79 for PA, 0.32 for PG, and 0.11 for ICA. **(A)** Surface-rendered 3D visualization generated in 3D Slicer, showing manual reference segmentations (left) and model predictions (right). Anatomical orientation markers indicate superior (S), inferior (I), right (R), and left (L). **(B)** Multiplanar and oblique views showing segmentation masks overlaid on the T1CE MRI volume. The axial and sagittal images are multiplanar reconstructions generated from the original coronal T1CE volume with the corresponding segmentation masks in 3D Slicer, not overlays on independently acquired axial or sagittal MRI sequences. PA, pituitary adenoma; PG, pituitary gland; ICA, internal carotid artery; DSC, Dice similarity coefficient; T1CE, contrast-enhanced T1-weighted.

**Figure 3** presents the best-performing segmentation case, with a mean DSC of 0.84 (0.94 for PA, 0.71 for PG, and 0.86 for ICA). In contrast, the worst performing case (**Figure 4**) exhibited a markedly lower mean DSC of 0.41, with corresponding values of 0.79, 0.32, and 0.11 for PA, PG, and ICA, respectively.

### 3.3 Exploratory subgroup analyses

Exploratory subgroup analyses revealed a consistent advantage of Swin-Unet over nnU-Net V2 in PG segmentation across acromegaly-related groups. For patients with acromegalic appearance, the mean DSCs were 0.697 ± 0.123 for Swin-Unet and 0.612 ± 0.150 for nnU-Net (uncorrected p = 0.008, d = 2.21). Similar trends were observed in diagnosed acromegaly (0.714 ± 0.095 vs. 0.659 ± 0.103; uncorrected p = 0.014, d = 1.51) and in GH-positive tumors (0.693 ± 0.090 vs. 0.648 ± 0.119; uncorrected p = 0.049, d = 0.77). In PA segmentation, nnU-Net exhibited superior segmentation performance compared to Swin-Unet in microadenoma cases (0.547 ± 0.045 vs. 0.418 ± 0.068; uncorrected p = 0.018, d = −4.29). For ICA segmentation in high T2 signal cases, Swin-Unet showed a marginal advantage (0.716 ± 0.181 vs. 0.693 ± 0.159; uncorrected p = 0.064, d = 0.41). As these findings are exploratory, they should be interpreted cautiously. Representative cases from the exploratory subgroup analyses are shown in **Figure 2**. **Figure 2A** presents a microadenoma case where nnU-Net outperformed Swin-Unet in PA segmentation. In contrast, **Figures 2B, C** show patients with diagnosed acromegaly, and **Figure 2D** features a patient with acromegaly, all demonstrating improved PG segmentation using Swin-Unet.

### 3.4 Slice-level recognition performance

Slice-wise recognition performance is summarized in **Table 3**. Both models demonstrated robust performance for PA and ICA segmentation with F1-score above 0.90, whereas PG segmentation showed comparatively more modest performance. Comparative analysis revealed that nnU-Net achieved slightly higher specificity for ICA and PG, while Swin-Unet yielded higher recall for PA and ICA.

**Table 3:** Performance of Swin-Unet and nnU-Net in slice-wise recognition of PA, PG, and ICA. Accuracy, precision, recall, and specificity are reported as mean values with 95% confidence intervals, and F1-scores are presented as mean values.

Mean [95% CI]	Accuracy	Precision	Recall	Specificity	F1-score
Swin-Unet					
PA	0.92 [0.89–0.94]	0.94 [0.91–0.96]	0.96 [0.93–0.98]	0.69 [0.55–0.79]	0.95
PG	0.79 [0.74–0.83]	0.75 [0.68–0.80]	0.86 [0.80–0.90]	0.73 [0.66–0.79]	0.80
ICA	0.87 [0.83–0.90]	0.90 [0.86–0.93]	0.94 [0.90–0.96]	0.58 [0.46–0.69]	0.92
nnU-Net					
PA	0.96 [0.95–0.97]	0.95 [0.92–0.97]	0.93 [0.90–0.96]	0.98 [0.96–0.99]	0.94
PG	0.91 [0.89–0.93]	0.77 [0.70–0.83]	0.78 [0.71–0.84]	0.94 [0.92–0.96]	0.78
ICA	0.94 [0.92–0.95]	0.95 [0.91–0.97]	0.86 [0.81–0.90]	0.98 [0.96–0.99]	0.90

### 3.5 Preprocessing and inference time

**Figure 5:**
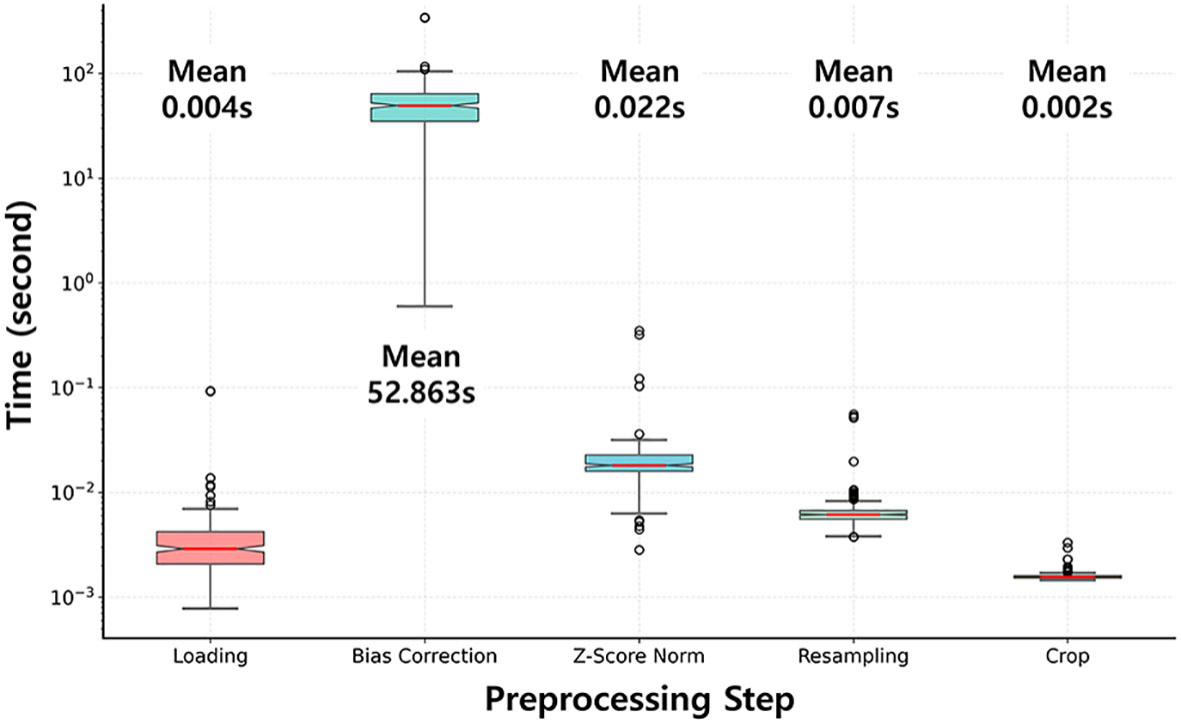
Complete distribution of preprocessing times for 255 patients across individual pipeline steps. Loading corresponds to data input, bias correction to N4 bias field correction, Z-score Norm to Z-score intensity normalization, resampling to isotropic spacing of 0.3 × 0.3 mm, and cropping to a centered 256 × 256 pixel region. Mean processing times are displayed for each preprocessing step. For Swin-Unet input preparation, fixed external resampling and cropping were employed. For nnU-Net, the standard self-configuring internal preprocessing pipeline was retained.

The mean preprocessing time per patient was 52.9 s [95% CI: 48.7-57.0], largely attributable to N4 bias correction, while all other preprocessing operations required less than 0.1 s. The complete distribution of preprocessing times across the dataset is presented in **Figure 5**. Inference and saving required 0.69 s [0.58–0.79] for Swin-Unet and 2.26 s [2.20–2.31] for nnU-Net. Consequently, the mean end-to-end processing times of 78.4 s [61.4–95.5] and 80.0 s [63.1–97.0] per patient for Swin-Unet and nnU-Net, respectively.

## 4 Discussion

This study investigated the feasibility of automated segmentation of the PA, PG, and ICA using two DL models, Swin-Unet and nnU-Net, applied exclusively to T1CE coronal MRI sequences. Both models demonstrated modest but comparable segmentation performance on overlap-based and boundary-based evaluation, with mean Dice scores of approximately 0.70 and 0.68, and overall HD95 values of approximately 5.21 mm and 5.30 mm for Swin-Unet and nnU-Net, respectively. Segmentation performance was highest for PA and ICA, whereas PG segmentation showed considerably lower scores and relatively higher boundary discrepancies. This performance discrepancy is largely attributed to the limited absolute volume size of the PG: even minimal boundary deviations produce substantial penalties in Dice similarity, reflecting the inherent challenge of achieving high voxel-level accuracy. Despite these limitations, slice-wise recognition provided more consistent performance, with F1-scores of 0.78–0.80 for PG and exceeding 0.90 for both PA and ICA. These findings indicate that while voxel-level PG delineation remains technically challenging, reliable slice-level identification of the gland, tumor, and carotid vasculature offers translational potential for clinical implementation.

Swin-Unet and nnU-Net revealed comparable performance despite their architectural differences. While Swin-Unet incorporates transformer mechanisms to capture long-range contextual information, nnU-Net implements a self-adaptive pipeline specifically optimized for biomedical image segmentation. Consistent with the DSC outcomes, HD95 values revealed no significant differential between the two architectures, indicating that neither architecture showed a meaningful advantage in voxel-wise overlap or boundary localization. Several factors may explain the observed performance convergence. Limited dataset size and acquisition heterogeneity potentially restricted the advantages typically associated with transformer-based modeling, alongside the robust baseline performance of nnU-Net. Our findings suggest complementary strengths between these approaches rather than clear superiority of either model.

In this study, Swin-Unet demonstrated a higher DSC than nnU-Net V2, though the difference was not statistically significant. Exploratory subgroup analyses indicated that Swin-Unet performed more effectively in acromegaly-related cases with enlarged and distorted pituitary anatomy, while nnU-Net showed better performance in patients with small, well-defined microadenomas. These findings suggest that anatomical complexity may significantly influence segmentation performance. Hybrid or adaptive approaches may improve robustness across diverse clinical scenarios.

The best-performing case demonstrated a mean DSC of 0.84 and mean HD95 of 2.51 mm across all structures, whereas the worst-performing case achieved only 0.41 and 9.21 mm, with particularly low accuracy for PG (0.33 and 6.47 mm) and ICA (0.11 and 17.12 mm). Despite presenting with a larger cystic adenoma, this worst-performing case exhibited impaired boundary delineation likely attributable to heterogeneous signal characteristics and Knosp grade 2 invasion. These findings indicate that segmentation performance is influenced primarily by lesion homogeneity and anatomical simplicity rather than tumor size, underscoring the need for further model optimization in cases with such complex morphological features.

Previous studies on pituitary tumor segmentation have predominantly focused on multi-sequence or multi-planar approaches, often reporting moderate accuracy under controlled, uniform imaging protocols (10–12). However, methods validated in controlled research settings may not reliably translate to clinical practice due to substantial variability in acquisition parameters across scanners and institutions (28). The present study addresses this limitation by restricting analysis to T1CE coronal MRI acquired under heterogeneous protocols. This approach reflects real-world clinical conditions, as T1CE coronal imaging is routinely available in most patients and accessible to centers with limited resources, underscoring its broader clinical applicability than multi-parametric protocols. To the best of our knowledge, this work represents one of the first systematic comparisons of transformer-based and CNN-based architectures applied to single sequence, enhancing the robustness and clinical relevance of our findings.

Computational efficiency represents a key advantage of this automated segmentation framework. The complete pipeline, including preprocessing and inference, executed in approximately 78 s [61–96], with inference alone requiring less than 1 s. This rapid processing performance supports practical integration into diverse clinical workflows and enables flexible deployment across diverse healthcare settings, ensuring rapid availability of 3D reconstructions for clinical assessment.

Several limitations should be acknowledged. First, segmentation accuracy at the voxel level was modest, particularly for the PG. Accurate delineation of the PG is inherently challenging given its small size, variable morphology, and frequent obscuration by adjacent adenomas on T1CE imaging. Second, this study was conducted at a single institution. Although scanner heterogeneity was maintained, the findings require external validation in multi-institutional cohorts with diverse patient populations to establish broader generalizability. Third, the analysis was restricted to T1CE coronal MRI. While this modality offers clinical practicality and is consistently available in routine pituitary protocols, incorporating additional modalities such as T2-weighted or dynamic contrast-enhanced imaging could provide complementary anatomical and functional information that may improve segmentation performance. Fourth, although two highly-experienced neurosurgeons cross-reviewed all manual annotations and achieved consensus using standardized structure-specific segmentation criteria, the absence of formal inter-rater and intra-rater reliability analyses represents a potential source of evaluation bias. Finally, the primary focus of this study was to establish technical feasibility rather than clinical integration, and further prospective validation studies are essential to demonstrate real-world clinical applicability.

## 5 Conclusion

Swin-Unet and nnU-Net were applied to coronal contrast-enhanced T1-weighted MRI for automated segmentation of the PA, PG, and ICA. Both models demonstrated modest segmentation accuracy on both overlap-based and boundary-based evaluation while consistently identifying these anatomical structures and generating approximate 3D reconstructions. While current segmentation precision remains inadequate for standalone clinical use, this single-sequence automated approach shows potential promise as a complementary tool for radiologic interpretation and preoperative surgical planning.

## Data Availability

The institutional data used for training and validation are not publicly available due to the protection of private patient health information.
