# Supplementary figures and images for: Automated segmentation of pituitary adenomas, pituitary gland, and internal carotid arteries on routine coronal contrast-enhanced T1-weighted MRI: a single-sequence feasibility study
Source: Front Endocrinol (Lausanne). 2026 Jun 30;17:1851379. doi: 10.3389/fendo.2026.1851379 (PMC13364558; doi:10.3389/fendo.2026.1851379)

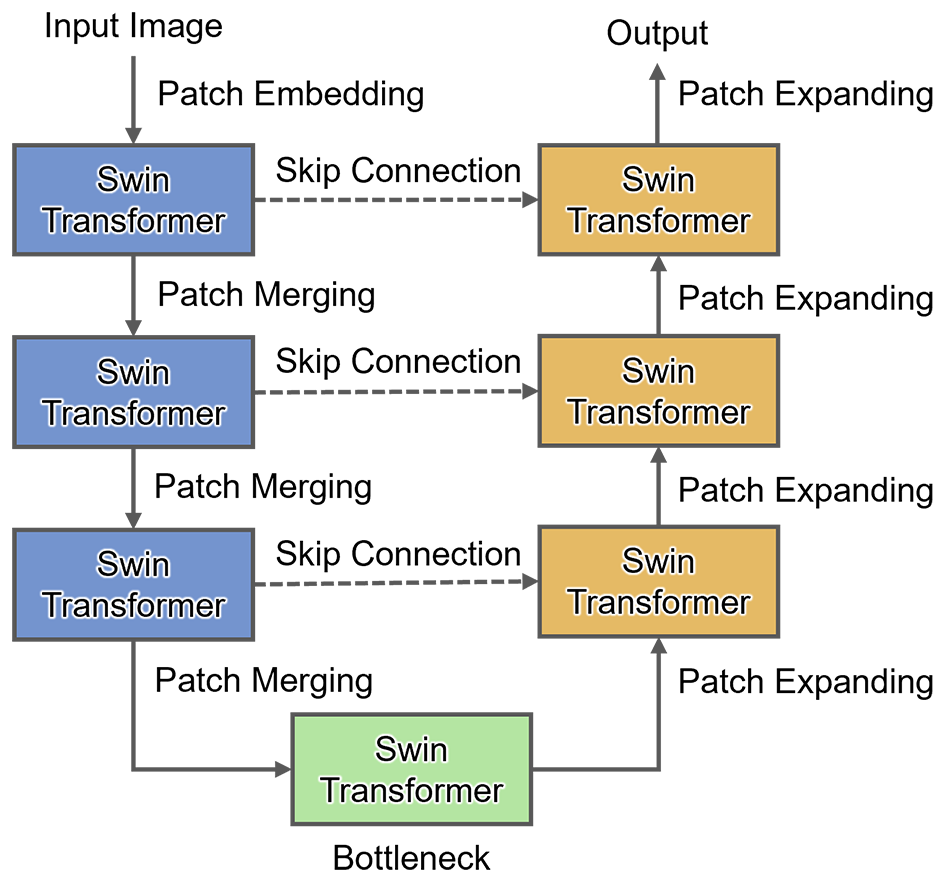

Supplement: Supplementary file 2 [file Image1.tif]

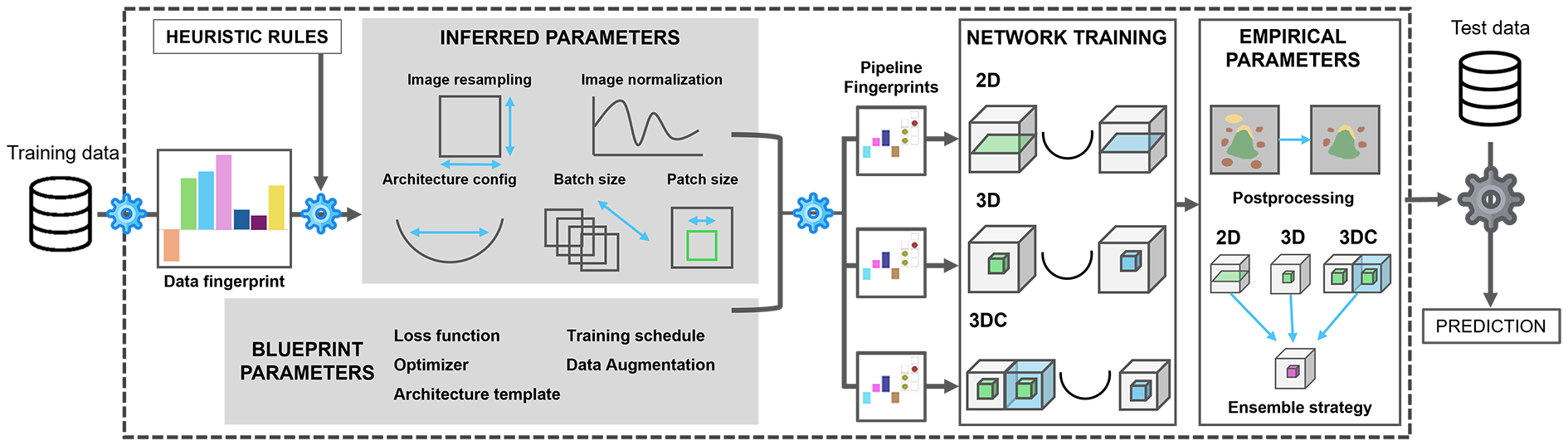

Supplement: Supplementary file 3 [file Image2.tif]
