# Supplementary material for: Automated segmentation of pituitary adenomas, pituitary gland, and internal carotid arteries on routine coronal contrast-enhanced T1-weighted MRI: a single-sequence feasibility study
Source: Front Endocrinol (Lausanne). 2026 Jun 30;17:1851379. doi: 10.3389/fendo.2026.1851379 (PMC13364558; doi:10.3389/fendo.2026.1851379)
Supplement: Supplementary file 5 [file DataSheet2.docx]

Supplementary Material

# Supplementary Figures and Tables

## Supplementary Figures

**Supplementary Figure 1.** The basic structure of the U-net–shaped transformer is composed of the encoder, bottleneck, decoder, and skip connections. The encoder, bottleneck, and decoder are constructed based on the Swin transformer.

**Supplementary Figure 2.** Flowchart of the nnU-Net pipeline. To ingest training data, nnU-Net uses heuristic rules to determine the data-dependent hyperparameters, referred to as “data fingerprint”. Inferred parameters, blueprint parameters, and data fingerprints produce pipeline fingerprints, which produce network training for 3D U-Net using the determined hyperparameters. The ensemble of network configurations, in combination with post-processing, determines the best average Dice coefficient for the training data. 2D, 2D U-Net; 3D, 3D U-Net; 3DC, 3D-Cascade U-Net.

# Supplementary Data

## MRI acquisition parameters

MRI acquisition parameters are provided in Supplementary Data Sheet 1 (CSV format).

## Supplementary Video

Representative 3D Slicer–based visualization of the segmentation outputs is provided in Supplementary Video 1.
